# Population Percentage and Population Size of Men Who Have Sex With Men in the United States, 2017-2021: Meta-Analysis of 5 Population-Based Surveys

**DOI:** 10.2196/56643

**Published:** 2024-06-11

**Authors:** Brady W Bennett, Stephanie DuBose, Ya-Lin A Huang, Christopher H Johnson, Karen W Hoover, Jeffrey Wiener, David W Purcell, Patrick S Sullivan

**Affiliations:** 1 Department of Epidemiology Rollins School of Public Health Emory University Atlanta, GA United States; 2 PRISM Health Research Group Rollins School of Public Health Emory University Atlanta, GA United States; 3 Division of HIV Prevention National Center for HIV, Viral Hepatitis, STD, and TB Prevention Centers for Disease Control and Prevention Atlanta, GA United States

**Keywords:** sexual behavior, sexual identity, sexual attraction, men who have sex with men, population estimates, MSM, men who have sex with other men, national surveys, census, United States

## Abstract

**Background:**

Male-to-male sexual transmission continues to account for the greatest proportion of new HIV diagnoses in the United States. However, calculating population-specific surveillance metrics for HIV and other sexually transmitted infections requires regularly updated estimates of the number and proportion of men who have sex with men (MSM) in the United States, which are not collected by census surveys.

**Objective:**

The purpose of this analysis was to estimate the number and percentage of MSM in the United States from population-based surveys.

**Methods:**

We used data from 5 population-based surveys to calculate weighted estimates of the proportion of MSM in the United States and pooled these estimates using meta-analytic procedures. We estimated the proportion of MSM using sexual behavior–based questions (encompassing anal or oral sex) for 3 recall periods—past 12 months, past 5 years, and lifetime. In addition, we estimated the proportion of MSM using self-reported identity and attraction survey responses. The total number of MSM and non-MSM in the United States were calculated from estimates of the percentage of MSM who reported sex with another man in the past 12 months.

**Results:**

The percentage of MSM varied by recall period: 3.3% (95% CI 1.7%-4.9%) indicated sex with another male in the past 12 months, 4.7% (95% CI 0.0%-33.8%) in the past 5 years, and 6.2% (95% CI 2.9%-9.5%) in their lifetime. There were comparable percentages of men who identified as gay or bisexual (3.4%, 95% CI 2.2%-4.6%) or who indicated that they are attracted to other men (4.9%, 95% CI 3.1%-6.7%) based on pooled estimates. Our estimate of the total number of MSM in the United States is 4,230,000 (95% CI 2,179,000-6,281,000) based on the history of recent sexual behavior (sex with another man in the past 12 months).

**Conclusions:**

We calculated the pooled percentage and number of MSM in the United States from a meta-analysis of population-based surveys collected from 2017 to 2021. These estimates update and expand upon those derived from the Centers for Disease Control and Prevention in 2012 by including estimates of the percentage of MSM based on sexual identity and sexual attraction. The percentage and number of MSM in the United States is an important indicator for calculating population-specific disease rates and eligibility for preventive interventions such as pre-exposure prophylaxis.

## Introduction

Men have been the epicenter of HIV infections in the United States since the beginning of the epidemic, accounting for the largest proportion of new cases each year [1]. Most new HIV infections are among gay, bisexual, and other men who have sex with men (MSM) [1]. In 2021, 67% of new HIV infections in the United States were among MSM [2]. To better understand national rates of HIV infections, medication adherence, and preventative measures among MSM, it is imperative to have accurate estimates of the proportion and number of MSM in the United States [3]. However, the most recent published national estimates for MSM populations were published over a decade ago [4].

There is currently no census-based question on same-sex behavior that yields data to estimate a national proportion or count of MSM in the United States, although there is 1 question about being part of a same-sex household [5]. However, these data exclude MSM who are not domiciled with a male partner or who decline to report that their housemate is a partner. Therefore, previous estimations of MSM in the United States have focused on periodically conducted representative surveys, such as the General Social Survey (GSS) [6] or National Health and Nutrition Examination Survey (NHANES) [7]. In 2012, Centers for Disease Control and Prevention scientists used meta-analysis [4] to determine the population size of MSM in the United States and calculate disease rates and rate ratios for HIV and primary and secondary syphilis [4]. Subsequent analyses by Grey et al [8] extended national estimates of MSM to smaller area estimates such as states and counties.

These past analyses focused on sexual behaviors to determine MSM status. Because some national surveys ask about sexual orientation or attraction, it also is possible to examine prevalence using orientation or attraction and consider the concordance between self-reported sexual behavior and self-reported sexual identity or attraction. Currently, the GSS, NHANES, and the National Survey of Family Growth (NSFG) [9] contain both sexual behavior and sexual identity or attraction questions. These 2 measures are not always concordant, and the concordance can vary by age and race [10,11]. It has been argued that for some public health uses of estimates such as estimating populations of MSM in need of HIV prevention services or testing, behavior might be a better indication of need than identity [8]. However, willingness to report a same-sex orientation may affect willingness to seek prevention services.

It has been over a decade since Purcell et al [4] published their national estimates of MSM, and in that time, it has been cited over 200 times [12]. However, there have also been major policy decisions, such as the 2015 Supreme Court decision affirming the right for same-sex couples to marry in *Obergefell v. Hodges* that may affect our understanding of the proportion of the population who are MSM. Since the landmark 2015 decision, public support for marriage equality has grown consistently year-over-year, indicating larger acceptance of lesbian, gay, bisexual, transgender, queer or questioning, intersex, and asexual persons [13]. Therefore, we sought to update national behavior prevalence estimates of MSM and explore whether there are enough data to provide estimates of MSM based on orientation or attraction. Following the methods of Purcell et al [4], we used population-based surveys and meta-analysis to estimate separate estimates of each survey and year combination and then aggregate estimates into single, nationally representative proportions of MSM in the United States.

## Methods

### Study Selection

To determine which population-based surveys to include in our analysis, we started with the selection methods used by Purcell et al [4]. We examined the sources used in the previous estimates to identify whether the data collection was ongoing. We then completed a literature search using PubMed (National Institutes of Health) and Google Scholar to identify additional sources that include questions about sexual behavior, sexual identity, or attraction. We searched using key terms for measurement (prevalence and estimation), male-to-male sexual behavior or identity (MSM, male-to-male sexual contact, gay, and bisexual), geography (United States), and survey (population-based survey). Abstracts were screened by the lead author (BWB) in consultation with the coauthors for data sources that used population-based surveys for estimation.

Studies eligible for inclusion were population-based surveys with available complex survey methodology documentation to allow weighting to obtain estimated population proportions of MSM and 95% CIs. Surveys that recruited predominantly at sites that are likely to have a high proportion of MSM (ie, surveys among HIV-infected persons and surveys at sexually transmitted infection clinics) were excluded to minimize overestimation. We identified 4 ongoing population-based surveys from Purcell et al [4] that currently include questions about sexual behavior, sexual identity, or attraction: the GSS [6], NHANES [7], NSFG [9], and National Survey on Drug Use and Health [14]. In addition to this, we also identified the National Health Interview Survey [15] as another eligible study (Table 1). Gallup [16] and the US Census Pulse Household Survey [17] also estimate the proportion of men who identify as gay or bisexual; however, we were unable to gain access to CIs for reported point estimates and, therefore, excluded these data from our analysis.

**Table 1:** Characteristics of eligible studies for meta-analysis on population size of MSM^a^ in the United States.

Study name	Population surveyed	Sampling method	Data collection periods	Recall periods	MSM questions category
General Social Survey	National household survey of the general US population of noninstitutionalized persons aged ≥18 years	2018: In-person probability sample of household in the United States 2021: phone and mail to web-based probability sample of households in the United States due to COVID-19. For both, 1 individual in each household completed the survey	2018 and 2021	Past year, past 5 years, and lifetime	Sexual behavior and sexual identity
National Health and Nutrition Examination Surveys	National household survey of the general US population of noninstitutionalized persons aged ≥12 years. Sexual behavior questions only asked of persons 18-59 years	Complex stratified, multistage cluster sample. Administration includes individual in-person interviews with representative sample of population	2017-2020	Past year and lifetime	Sexual behavior and sexual identity
National Household Survey on Drug Use and Health	National household survey of the general US population of noninstitutionalized persons aged ≥12 years. Sexual behavior questions only asked of persons aged 18-59 years	Complex stratified, multistage cluster sample; administration through individual in-person interviews with representative sample of population	2018, 2019	N/A^b^	Sexual identity and sexual attraction
National Survey of Family Growth	National household survey of the general US population of noninstitutionalized persons aged 15-44 years. Oversampling of Black and Hispanic adults	A nationally representative multistage area probability sample. Conducted through individual in-person interviewing. Sensitive questions answered privately by self-administration using computer-assisted personal interviewing	2017-2019	Past year, lifetime	Sexual behavior, sexual identity, and sexual attraction
National Health Interview Survey	National household survey of the general US population of noninstitutionalized persons aged 18 years and older	Continuously collected complex stratified, multistage cluster sample using computer-assisted personal interviewing	2019	N/A	Sexual identity

^a^MSM: men who have sex with men.

^b^N/A: not applicable.

### Survey-Specific MSM Estimation

We analyzed data from the most recent survey years available at the time of analyses to estimate the numbers of MSM. NHANES, which collects survey data in 2-year increments, created a combined data set from 2017 to March 2020 to account for the disrupted collection period in 2020 due to COVID-19. We use this data set rather than 2017-2018 to allow for the inclusion of the most up-to-date data years. No other data set included data from 2020.

To determine the study-level prevalence of MSM by sexual behavior, we considered questions about anal or oral sex with another male for 3 time periods: lifetime, in the past 5 years, and in the past 12 months. Survey-specific questions are outlined in Table S1 in Multimedia Appendix 1. For sexual identity, we categorized men who indicated that “gay,” “homosexual,” or “bisexual” best described them to be MSM. For sexual attraction, we considered men who indicated that they are “equally attracted to males and females,” “mostly attracted to the same sex,” or “only attracted to the same sex” to be MSM. Data were analyzed accounting for the complex sample design [18]; proportions of MSM and variances were estimated for each behavioral domain, for attraction and for orientation.

### Meta-Analysis

We applied Rao et al’s [19] meta-analytic method to pool survey-specific results into a single estimate with confidence bounds. First, for each recall period (eg, lifetime sex, past 5 years, and past 12 months) and for each identity or attraction estimate, we multiplied the population-level prevalence by the inverse of its variance. Then, we summed these weighted prevalence estimates across studies and then divided by the sum of the weights. Because surveys included in our analysis were conducted over several years and with differing sample designs and age ranges, we included a corresponding between-studies variance term [20] before deriving the overall prevalence estimates.

We examined heterogeneity of prevalence estimates across surveys using the Q-statistic [19] and Higgins I index [21]. Pooled estimates for the overall prevalence of MSM were based on random-effects models, which provide a more conservative estimate of the variance, generating potentially more accurate inferences about a population of studies beyond what we present in this analysis [4]. All meta-analytic calculations were completed in R (R Foundation for Statistical Computing) using the “meta” package [22].

### Calculation of Count of MSM and Other Men in the United States

To calculate the total number of MSM and non-MSM, aged 18 years and older, in the United States, we took our recently derived past 12-month estimate of the proportion of MSM and its 95% CI and multiplied it by the 2022 population estimate of men aged 18 years and older in the United States from the US Census Bureau [23]. The number of MSM was then subtracted from the total estimated number of men aged 18 years and older in the United States to compute the population size of other men.

### Ethical Considerations

This project did not require review by an internal review board because it did not include human subjects, nor was it a clinical investigation as defined by federal regulations [24].

## Results

### MSM Estimates Based on Sexual Behavior

The estimated proportions of men who have sex with other men, by recall period, for each of the included population-based surveys that contained sexual behavior–based questions and the pooled estimates obtained from the meta-analysis are presented in Table 2. The pooled estimates were 3.3% (95% CI 1.7%-4.9%) in the last 12 months, 4.7% (95% CI 0.0%-33.8%) in the last 5 years, and 6.2% (95% CI 2.9%-9.5%) in their lifetime. The tests for heterogeneity were significant for both the “past 5 years” and “lifetime sex” recall periods but not for the past 12 months recall period.

**Table 2:** Estimated proportion of men who have sex with men for individual studies and combined meta-analysis by behavior, NSFG^a^, NHANES^b^, and GSS^c^ surveys, United States, 2017-2021.

Study name		Time period	Sample size	Estimated prevalence (%; 95% CI)		*Q* statistic^d^		*P* value		*I*^2^ (%)^e^	
**Past 12 months**					3.3 (1.7-4.9)		6.8		.08		55.6
	GSS	2018	502	2.9 (1.4-4.4)							
	GSS	2021	809	5.0 (3.3-6.7)							
	NSFG	2017-2019	5206	3.1 (2.4-3.8)							
	NHANES	2017-2020	3338	2.7 (1.9-3.4)							
**Past 5 years**					4.7 (0.0-33.8)		14.4		<.001		93.1
	GSS	2018	559	2.5 (1.2-3.8)							
	GSS	2021	904	7.1 (5.1-9.1)							
**Lifetime sex**					6.2 (2.9-9.5)		25.9		<.001		88.4
	GSS	2018	500	3.1 (1.6-4.6)							
	GSS	2021	807	7.8 (5.6-10.0)							
	NSFG	2017-2019	5163	7.0 (6.9-7.0)							
	NHANES	2017-2020	3338	6.9 (5.6-8.2)							

^a^NSFG: National Survey of Family Growth.

^b^NHANES: National Health and Nutrition Examination Survey.

^c^GSS: General Social Survey.

^d^*Q*-statistic follows a chi-square distribution to determine the presence or absence of heterogeneity in a set of studies in a meta-analysis.

^e^*I*^2^ quantifies the degree of heterogeneity in a meta-analysis.

### MSM Estimates Based on Sexual Identity and Sexual Attraction

Table 3 contains the estimated prevalence and 95% CIs, along with the pooled aggregate results, by self-reported sexual identity and attraction. The pooled estimated prevalence of gay and bisexual men by sexual identity was 3.4% (95% CI 2.2-%4.6%), which comes from 8 collection cycles across 5 different surveys. Both tests for heterogeneity (*Q*-statistic and *I*^2^ indicated heterogeneity across surveys (*I*^2^=95.4%; *Q*=152.4; *P*<.001). The pooled estimated prevalence of gay and bisexual men by sexual attraction was 4.9% (95% CI 3.1%-6.7%), with significant heterogeneity across the 3 collection periods from 2 surveys (*I*^2^=79.2; *Q*=9.6; *P*=.008). We calculated pooled estimates of the proportion of gay and bisexual men by including a broader definition that includes men who responded that they are “mostly attracted to women.” This broader definition resulted in a proportion of MSM of 9.3%, which is substantially higher than any other proportion calculated from this analysis. Therefore, for the purpose of this paper, we used the stricter definition of MSM based on attraction (ie, men who indicated that they are “equally attracted to males and females,” “mostly attracted to the same sex,” or “only attracted to the same sex”).

Our data indicate limited fluidity in respondents’ identification and attraction. For the GSS, which has both sexual identity or attraction questions and sexual behavior questions, approximately 0.6% of straight-identifying men in 2018 sample and 2.6% in the 2021 sample also reported anal or oral sex with another man in the past 5 years. Similarly, for the 2017-2019 NSFG, of men who identified as straight, 4.4%-5.9% also reported anal or oral sex with another man in their lifetime and 0.9%-2.2% reported anal or oral sex in the past 12 months.

Table 4 shows the estimated number of MSM and non-MSM men aged 18 years and older in the United States. These totals were derived by multiplying the proportion of MSM from the pooled estimate for sexual behavior in the past 12 months (95% CI 1.7-4.9) by the total number of men aged 18 years and older in the United States [25]. We estimated that there were 4,230,000 MSM (95% CI 2,179,000-6,281,000) aged ≥18 years in the United States.

**Table 3:** Estimated proportion of men who identify as gay or bisexual for individual studies and combined meta-analysis by identity and attraction (GSS^a^, NSDUH^b^, NSFG^c^, NHANES^d^, and NHIS^e^ surveys, United States, 2017-2021).

Study name			Time period		Sample size		Estimated prevalence (%; 95% CI)		*Q* statistic		*P* value		*I*^2^ (%)
**Identity**							3.4 (2.2-4.6)		152.4		<.001		95.4
	GSS	2018		629		2.2 (1.1-3.2)							
	GSS	2021		1012		6.4 (4.7-8.0)							
	NSDUH	2018		20,162		4.6 (4.1-5.0)							
	NSDUH	2019		19,923		4.2 (3.8-4.6)							
	NSFG^f^	2017-2019		5206		2.5 (1.9-3.2)							
	NSFG^f^	2017-2019		5206		2.6 (1.9-3.3)							
	NHIS	2019		14,733		2.1 (1.8-2.4)							
	NHANES	2017-2020		2595		4.6 (3.5-5.7)							
**Attraction**							4.9 (3.1-6.7)		9.6		.008		79.2
	NSDUH	2018		20,162		5.3 (4.8-5.7)							
	NSDUH	2019		19,923		5.3 (4.9-5.7)							
	NSFG	2017-2019		5206		4.0 (3.2-4.8)							

^a^GSS: General Social Survey.

^b^NSDUH: National Household Survey on Drug Use and Health.

^c^NSFG: National Survey of Family Growth.

^d^NHANES: National Health and Nutrition Examination Survey.

^e^NHIS: National Health Interview Survey.

^f^The NSFG contains 2 different questions about a respondent’s sexual identity; therefore, we included both estimates for 2017-2019.

**Table 4:** Population size of men aged 18 years and older in the United States, 2022 and number of MSM^a^ and non-MSM using past 12 months proportion estimate of MSM from meta-analysis.

	MSM proportion (%)	MSM population size, n	Non-MSM population proportion (%)	Non-MSM population size, n
Population estimation (95% CI)	3.3 (1.7-4.9)	4,230,000 (2,179,000-6,281,000)	96.7 (95.1-98.3)	123,945,000 (121,894,000-125,996,000)

^a^MSM: men who have sex with men.

## Discussion

### Principal Findings

Our meta-analysis of data from 5 population-based surveys indicated that 3.3% of the US male population report recent sex with men, and 3.4%-4.9% of the US male population report identifying as gay, bisexual, or being sexual attracted to men. We found substantial overlap between estimates based on behavioral measures and measures based on orientation or attraction. Our behavioral estimates are all within 1 percentage point of the Centers for Disease Control and Prevention’s 3 behavior estimates from over a decade ago [4]. Additionally, compared to this analysis, they include more national surveys that asked about sexual behavior or identification [4]. Although there is growing interest in understanding the experiences and risks of MSM, there is a smaller number of ongoing population-based studies that examine these issues.

Previous estimates by Purcell et al [4] have also been used extensively in calculating the rates of disease among MSM. We hope that these updated percentages of MSM will be used in future analyses to calculate the rates of health states and conditions among MSM. For example, time periods for syphilis diagnoses can be matched to time periods for the estimated MSM population size. Then, the count of syphilis diagnoses among MSM can be divided by the estimated population size of MSM for that same recall period and transformed into rates per 100,000 population.

For our calculation of the total number of MSM in the United States, we chose to use the prevalence of male-male sex during the past 12 months. This differs from the previous paper by Purcell et al [4], which used the estimated prevalence from self-reported sex with a man in the past 5 years. Our decision was both practical and methodical. *Q*-statistics and *I*^2^ for the pooled “past 5 years” estimates indicated heterogeneity across surveys (*Q*=14.4; *P*<.001; *I*^2^=93.1%). In addition, our “past 5 years” estimates also had very wide 95% CIs (0.0-33.8), indicating a substantial amount of instability in the estimate. Out of the behavior-based estimates in our analysis, self-reported sex in the past 12 months had the lowest heterogeneity. In addition to considering current risk for sexually transmitted infections, using an estimate based upon recent sexual activity may allow us to enumerate those MSM who are currently most at risk for disease acquisition or most appropriate for prevention services.

Estimates from “past 5 years” or “lifetime” behavioral data might be used in other circumstances. For example, Purcell et al [4] use of the “past 5 years” measure to estimate number of MSM in the United States in 2012 was designed to include a broader section of MSM, including those who were not recently sexually active. Such denominators might be relevant for characterizing the impact of MSM on other types of diseases, depending on the timelines for exposure and pathogenesis. Having separate behavioral measurements with different recall periods allow users to determine which estimate makes the most sense for their public health purposes.

We expanded on previous work by calculating the prevalence of self-reported sexual identity and attraction to update and to understand the percentage of MSM in the United States. Identity and attraction are neither mutually exclusive nor perfectly concordant, and they do not necessarily overlap with behavior. The term MSM specifically refers to sexual behavior, but identity and attraction refer to how men classify themselves—an important distinction for epidemiologists and sexual health researchers who seek to avert disease transmission associated with male-male sex. Identity and attraction are not risk factors for sexually transmitted infections but may be proxies for current or future risks and for prevention service needs. However, as our data show, identity and attraction are not necessarily specific or sensitive [26]: a person who identifies as straight may still engage in anal or oral sex with another man, a man identifying as gay or bisexual may have anal or vaginal sex with a female. Providing additional prevalence estimates for orientation and attraction gives users more choices to fit their needs when trying to estimate these proportion of men who identify as gay or bisexual or are attracted to men, based on their programmatic goal or research question. Furthermore, including this new estimate of sexual identity and attraction aligns with new reports form the US Census Bureau, which began collecting data on sexual orientation in 2021. According to these data, 6.5% of cisgendered US men reported identifying as gay or bisexual [27].

Here, we calculated the 3 updated prevalence estimates for MSM, a new estimate for gay or bisexual men, and a new estimate for men who are attracted to other men. Going forward, we plan to update these estimates at regular intervals and to share public use data sets through AIDSVu [28]. We propose to update these estimates every 5 years, given the pace at which the national population-based surveys are implemented and published. We do not anticipate a substantial change in the estimates in the coming 5 years. Comparing the newly calculated “past 12 months” estimate to the “past 12 months” estimate from Purcell et al [4], we found a relatively small change over a decade of time (3.3% vs 2.9%, respectively). However, the visibility of sexual behavior and identity in our culture has shifted rapidly over the past few decades, and regular updated analyses will allow us to identify any unexpected shifts should they emerge and to provide estimates that always include the most recently collected data.

### Limitations

This analysis has important limitations. First, meta-analyses rely on the strengths of the underlying studies. The surveys that we used are minimally biased in terms of sampling—5 population-based surveys—but they have varying sample sizes of MSM (which comprise a small subpopulation of the sampled population). Thus, the individual survey estimates that feed into our pooled estimates have varying confidence limits around their point estimates. Furthermore, these surveys have varying age ranges for inclusion in the survey and thus may not be exactly transferable across surveys. Similar to Purcell et al [4], we were unable to directly calculate stratified estimates of the proportion of MSM by race, ethnicity, or age because of small sample sizes within stratifications.

There is also the potential for misclassification of a respondent’s true sexual behavior, identity, or attraction, leading to an underestimation of the count and percentage of MSM in the United States from our calculation. A recent analysis in Canada found that 13.5% of the gay and bisexual male population (based upon sexual behavior) reported being unlikely to disclose their sexual identity on government surveys [29]. However, it is unclear whether these estimates of misclassification are transferrable to the US population. Additionally, although we provide estimated percentages of MSM by sexual attraction and sexual identity, our final estimation of the total MSM population for the United States was solely based on sexual behavior (anal or oral sex) in the past 12 months rather than identity or attraction alone or a combination of identity or attraction and behavior.

Finally, we did not adjust for differing age ranges between individual surveys. This may have implications for our outcomes; however, only 1 survey, the NSFG, included men younger than 18 years in their estimate, and the number of respondents younger than 18 years old who answered that they had ever had sex with another man was <0.5% of the total respondents for NSFG and is, therefore, unlikely to have a significant impact on the results. Only the NSFG included data from men aged younger than 18 years; all other estimates for sexual behavior or identity or attraction used men older than18 years of age.

### Conclusions

The National HIV or AIDS strategy and the Ending the HIV Epidemic Initiative in the United States each highlight the prevention of HIV among MSM as key to meeting the goal of decreasing new HIV infections by 90% by 2030 [30]. Developing updated, accurate estimates of this population at risk is critical to better understanding the disproportionate burden of HIV and risk for HIV among MSM. Updated estimates of the MSM population sizes can help to guide resource allocation and programmatic efforts and support key benchmarks for progress as we seek to end the HIV epidemic and other health concerns that disproportionately impact MSM. Providing public, updated estimates of behaviors across different time periods and data on orientation and attraction can offer choices to researchers and health departments seeking to serve these populations.

## Appendix

Multimedia Appendix 1 Survey-specific questions.

## Abbreviations

GSS: General Social Survey
MSM: men who have sex with men
NHANES: National Health and Nutrition Examination Survey
NSFG: National Survey of Family Growth

## Data Availability

Data from General Social Survey, National Survey of Family Growth, National Health Interview Survey, and National Household Survey on Drug Use and Health are publicly available at their respective survey websites [4,6,14,15]. The 2017 to 2020 National Health and Nutrition Examination Survey sexual behavior data are not publicly available due to being “limited access data,” containing sensitive private information, but they can be accessed at a National Center for Health Statistics Research Data Center [7].
