# Supplementary material for: Population Percentage and Population Size of Men Who Have Sex With Men in the United States, 2017-2021: Meta-Analysis of 5 Population-Based Surveys
Source: JMIR Public Health Surveill. 2024 Jun 11;10:e56643. doi: 10.2196/56643 (PMC11200033; doi:10.2196/56643)
Supplement: Multimedia Appendix 1 [file publichealth_v10i1e56643_app1.docx]

**Literature Search Terms**

Estimation AND (men who have sex with men OR male-to-male sexual contact OR gay OR bisexual) AND population-based surveys AND United States

**Table S1.** Survey indicators, questions, and answer choices

| **Study Name** | **Indicator** | **Question** | **Answer Choices** |
| --- | --- | --- | --- |
| General Social Survey | Sex in the past year | Have your sex partners in the past year been… | Exclusively male; Both male and female; Exclusively female; Don't know; No answer; Not applicable |
|  | Sex in the past 5 years | Now thinking about the past five-years, have your sex partners int eh past five years been… | Exclusively male; Both male and female; Exclusively female; Don't know; No answer; Not applicable |
|  | Sex ever | Calculated from Sex in the past 12 months and Sex in the past 5 years | If respondent was categorized as having sex with a male in the past 5 years or as having sex with a male in the past 12 months, then they were categorized as having had sex with another man “ever” |
|  | Sexual Identity | Which of the following best describes you? | Gay, lesbian, or homosexual; Bisexual; Heterosexual or straight; Don't know; No answer; Not applicable; Skipped on web |
| National Health and Nutrition Examination Survey | Sex ever | Have you ever had any kind of sex with a man, including oral or anal? | Yes; No; Refused; Don't know |
|  | Sex in the past year | In the past 12 months, with how many men have you had anal or oral sex? | Range of numerical values |
|  | Sexual Identity | Which of the following best represents how you think of yourself? | Gay; Straight - that is not gay; Bisexual; Something else; I don't know the answer; Refused; Don’t know |
| National Survey of Family Growth | Sex in the past year | Calculated from number of sexual partners in the past 12 months | If respondent had sex with ≥1 person in the past 12 months, then categorized as having sex with another man in the past 12 months. |
|  | Sex ever | Have you ever had oral or anal sex with a male? | Yes; No; Not ascertained; inapplicable |
|  | Sexual Identity | Do you think of yourself as: | Heterosexual or straight; homosexual or gay; bisexual; not ascertained; refused; don't know |
|  | Sexual Attraction | People are different in their sexual attraction to other people. Which best describes your feelings? Are you… | Only attracted to females; mostly attracted to females; equally attracted to females and males; mostly attracted to males; only attracted to males; not sure; not ascertained |
| National Survey of Drug Use and Health | Sexual Identity | Which of the following do you consider yourself to be? | Heterosexual, that is, straight; Lesbian or gay; Bisexual; Don't know |
|  | Sexual Attraction | People are different in their sexual attraction to other people. What best describes your feelings | I am only attracted to opposite sex; I am mostly attracted to opposite sex; I am equally attracted to males and females; I am mostly attracted to same sex; I am only attracted to same sex; I am not sure |
| National Health Interview Survey | Sexual Identity | Do you think of yourself as: | Gay/lesbian; Straight, that is not gay/lesbian; bisexual; something else; I don't know the answer |
